# Modern Sociology

**Published:** 1900-11-17

**Authors:** 


### Modern Sociology.

### DANGEROUS TRADES

### Dust and Disease.

The connection between dust and disease has often been noted; and even the ordinary dust of the street and the household carries insidious dangers; but these are not so sure in their action as those caused by the dust raised in various manufacturing processes. Take, for example, the work of paper-staining. In old days there was a great fancy for what were called "flock" papers; they were regarded as handsome and comfortable-looking; and after a long period of neglect flock papers are beginning again to come into fashion. Fashion is thoughtless rather than cruel in her fads, and therefore it may be well to point out how the process of "flocking" is carried out. The paper having had the pattern outlined on it, the parts to be flocked are covered with a thin coat of size, and are then dusted over with the flock—a sort of felt dust—which is shaken out of something like a pepper-castor. This flock adheres to the parts that are coated with size, and when it has dried upon them the remainder is shaken off. All through the process dust is in evidence, penetrating to the worker's nostrils and lungs, irritating the membranes and causing disease.

Very similar to this is the process of bronzing, as applied to paper or leather. Here also the powder is dusted on to the prepared surface, generally by means of a pad, and the worker is exposed to the dangers of inhaling it. The committee on dangerous trades was asked to inquire into the use of metallochrome powder in lithographic works at the same time as bronzing; but on inquiry they were frequently assured that metallochrome powder was unknown. The fact was that those who used it did not know it under that name. When inquiry was made as to the employment of "flake white" or "Chinese white" information was readily forthcoming. It must not be assumed, however, that all the substances known by these names are identical in composition. On the contrary, some forms of flake white are quite harmless; while others are, to the extent of more than half, composed of white lead, the remainder being French chalk. One firm which had used this compound, finding that some cases of illness resulted among those employed, gave it up, and employed instead a powder called "metallochrome," which was composed of sulphate of barium, and was quite free from lead. This change did away with one important danger for the workers in this industry—which is, fortunately, a very small one—that of lead poisoning.

The use of flake white is to give depth and body to other colours—red especially being improved by it—besides giving tone and solidity to white tints themselves. It is chiefly used in transfers for printing the arms of railway companies on their coaches, these transfers being also required by brewers, tobacconists, and others, for such purposes as advertisements on tin trays, or decorations and trade marks on boxes. The flake white powder is either shaken over the transfer or it is dusted on with a soft pad, in which case the dust rises with each application of the pad. When the dust is more than half white lead, it is peculiarly dangerous to health; but even without absolutely poisonous stuff being included in it the mechanical irritation to throat, lungs, and nostrils is sufficient to condemn it. It is a trade at which no one seems to work long, though, as it is light work, there is no difficulty in filling up such vacancies as arise. Flake white is also used as an ingredient for printers' ink; but then it is in a liquid state, and in this condition little danger attends its use. In some lithographic firms also flake white is employed in a liquid state. The use of it in this way implies no danger, and the committee expressed the hope that, whenever possible, the printers and transfer-makers would adopt this method of manufacture.

Among other dusty and dangerous industries may be noted the manufacture of basic slag. Basic slag is the refuse that is left in the converter after making steel by the "Gilchrist Thomas" process, and is valuable as a manure. In order to be used for this purpose, however, the slag, which comes out of the converter in large pieces, must be ground into a powder so fine that from 80 to 85 per cent. of it would pass through a mesh of 10,000 to the square inch. Sometimes, if very dry, the slag is moistened before being ground; but in spite of this, much dust arises in the process of repeated grinding, which is necessary to reduce the slag to powder. Time after time is the process repeated, the fine dust being carried away by a fan to hoppers, from which it falls into bags without being touched by hand, while the heavier dust is carried into a chamber which must be cleared every six weeks. The men who perform this duty wear respirators. The dust given out by the slag in process of grinding is not in itself of a poisonous nature, but it causes, by its mechanical irritation, huskiness, asthma, and even pneumonia. It is chiefly in the packing of the dust that the workmen encounter danger, though this may be kept in check by the rejection of old or unsuitable bags. In one factory, only new jute bags are employed. A peculiar case occurred some time ago at Ellesmere Port. A labourer, not employed in any mill, but only in unloading bags of basic-slag dust, died of pneumonia, and at the inquest it came out that the disease had arisen from inhaling the dust. It is evident that respirators or veils should be used by those employed at every stage of the process.

Silicate of cotton is another slag product, in the making of which much dust arises. The process by which it is made is simple and ingenious. A fine stream of molten slag is met on issuing from the furnace by a strong blast of steam. By this, small globular particles of slag are formed, and are driven with great velocity into a chamber; from each of these particles, by reason of their viscous nature and the velocity with which they are forced through the air, is drawn a long thin filament. These filaments combine into a threadlike, spongy material, the silicate of cotton—or, as it is generally called, slag wool. This material is absolutely non-inflammable, and is a non-conductor of both heat and of sound. It is thus of great use in packing the pipes from steam-boilers, engines, &c., for laying between floors to deaden the sound, to render walls and floors fireproof, and very largely for cold storage purposes. In appearance it resembles cotton wool, but has no elasticity. It is very light. The dangers that accompany the manufacture are, again, the risk of inhaling particles of the slag wool while packing it. The fine particles of wool cause great irritation to skin and nose to a stranger, though it is said that the workers themselves get quite accustomed to it. It has been shown, however, that the irritation has caused several persons to give up the work. The employment of crape or muslin veils has been found to afford great relief from these symptoms.

